# Identifying diversity, equity, and inclusion enhancement opportunities through an online mixed methods library survey

**DOI:** 10.5195/jmla.2022.1436

**Published:** 2022-10-01

**Authors:** Jane Morgan-Daniel, Hannah F. Norton, Lauren E. Adkins, Michele R. Tennant, Mary E. Edwards, Matthew Daley

**Affiliations:** 1 morgandanie.jane@ufl.edu, Community Engagement and Health Literacy Librarian, University of Florida Health Science Center Libraries, Gainesville, FL.; 2 nortonh@ufl.edu, Chair, University of Florida Health Science Center Libraries, Gainesville, FL.; 3 lauren.adkins@ufl.edu, Pharmacy Liaison Librarian, University of Florida Health Science Center Libraries, Gainesville, FL.; 4 tennantm@ufl.edu, Interim Senior Director, Academic Research Consulting and Services (ARCS), University of Florida Health Science Center Libraries, Gainesville, FL.; 5 meedwards@ufl.edu, Liaison and Reference Librarian, University of Florida Health Science Center Libraries, Gainesville, FL.; 6 daleym@ufl.edu, Webmaster/Designer, University of Florida Health Science Center Libraries, Gainesville, FL.

**Keywords:** Diversity, equity, inclusion, mixed methods, online surveys, health science libraries, medical libraries

## Abstract

**Objective:**

A mixed methods survey was conducted at a health sciences library to assess patrons' perceptions of the library's digital and physical environments in relation to diversity, equity, and inclusion (DEI).

**Methods:**

Developed by the library's DEI Team and preceded by a pilot assessment, the survey posed 17 Likert Scale questions and 2 free-text response questions on the topics of belonging, inclusivity, equitability, emotional and physical safety, and commitment to DEI. The survey was created in Qualtrics, pilot tested, and launched in February 2020 for approximately 12 weeks.

**Results:**

Objective question responses were received from 101 individuals, with 24 open-ended responses. The quantitative findings showed largely positive perceptions of the DEI climate. Questions about feeling welcome and feeling physically safe were among those with the highest responses. The three lower-scoring questions indicate areas for improvement, including services for people whose native language is not English, for individuals with disabilities, and for families. The qualitative findings indicate the library's strengths include its exhibitions, welcoming atmosphere, and LGBTQ+ inclusivity initiatives. In contrast, opportunities for enhancement encompass non-English language resources, website updates, and accessibility to some physical spaces.

**Conclusion:**

The DEI Team is using the online survey data to enhance library services, staffing, programming, policies, and spaces. These improvements include looking into providing a space for patrons with families, expanding services for individuals whose first language is not English, assessing library accessibility for people with physical disabilities, and enhancing the physical space with quiet areas, improved lighting, and meditation spaces. Employee DEI training is ongoing, using results from a training needs survey to identify knowledge gaps. The library has a history of successful partnerships with campus entities, which will help the DEI Team to move forward with their work.

## INTRODUCTION

The University of Florida's (UF) libraries are committed to broadening access to information resources and establishing a welcoming, respectful, and inclusive environment for its 57,000 students, its employees, and the public, with a focus on underserved populations, consistent with the values of the library profession and UF's mission. Integrating Diversity, Equity, and Inclusion (DEI) principles into libraries' everyday practice, “rejecting the notion of libraries as neutral spaces,” and continually assessing structural and professional norms is essential “in order to have an inclusive, integrated, and involved library where all workers and patrons feel welcomed, are valued, and are treated equitably” [1-2].

Surveying attitudes towards library services and whether patrons' needs are met is common practice within health sciences librarianship [3]. DEI climate surveys for patrons are far less common, with most focusing on library employees' perceptions and needs relating to organizational climate, for example ClimateQUAL [4].

In 2019, UF's Health Science Center Libraries (HSCL) designed a multi-phase needs assessment, beginning with a pilot survey known as “HappyOrNot” [5]. Two HappyOrNot feedback terminals were placed in the HSCL to gather data on patrons' perceptions of the existing DEI climate [5]. The machines displayed 1 DEI-related question per week between April 12 - July 8, 2019, to which participants responded by pressing a button to indicate whether they were “very happy”, “happy”, “unhappy”, or “very unhappy”. Twelve closed-ended questions were posed in total and 3,445 responses were received. Additionally, 7 open-ended comments were gathered through a box placed by each machine. The survey questions developed for the HappyOrNot pilot were largely inspired by university-level DEI climate surveys found through a literature search, including Case Western Reserve University's Climate Survey and the University of Michigan's Campus Climate Survey on Diversity, Equity and Inclusion [6, 7]. Unfortunately, the project team was unable to use climate survey information from the University of Florida at large, as the questions and data from the last institutional survey conducted in 2015 are no longer available. Furthermore, although ClimateQUAL has previously been conducted for UF library employees, no similar survey has been carried out for UF library patrons. An additional source consulted when planning the HappyOrNot pilot was the Medical Library Association's 2019 membership-level DEI survey [8]. Three questions pertaining to respect, commitment to DEI, and whether the environment is welcoming were added based on this. The HappyOrNot terminals allowed HSCL to pose targeted DEI questions to collect immediate, anonymous feedback from our wide demographic of in-person clients. However, three significant drawbacks of this methodology were the inability to gather data on participants' specific demographics, the fact that people who do not use the HSCL in-person could not participate, and that the open-ended data collection process was less than optimal as it was not integrated within the feedback terminals. More detailed data was therefore needed to explore whether underrepresented groups perceived the library's DEI climate differently to their majority counterparts.

To build on the pilot's findings, HSCL launched an online survey in February 2020 to explore patrons' perceptions of the library's digital and physical environments in relation to DEI. The team anticipated that the survey's results would mirror findings from the literature, in which participants with historically marginalized identities tended toward less positive DEI climate experiences [6, 7].

## METHODS

The design of the 2020 online survey was influenced by the findings of the 2019 pilot study. To address pilot limitations, the Team developed the online survey with open and closed-ended questions, to include those who do not physically come to the library, and with demographic questions to facilitate detailed analysis of specific patron groups. The online survey therefore included the original HappyOrNot questions, augmented with demographic questions and new queries related to the inclusiveness of HSCL's web presence, as well as the library's inclusiveness to families, diverse religions, and other identities. Five further questions focused on imposter syndrome, belonging, religious and spiritual practice, and digital inclusivity. Imposter syndrome, which may include feelings of being a fraud or not deserving one's accomplishments, has been well-documented among students, including those with non-traditional paths to college, racial and ethnic minorities, and first-generation students [9]. Imposter syndrome has also been reported among health sciences librarians [10]. Regarding religious and spiritual practice, a survey of 351 medical students at the University of Florida's College of Medicine found that 54% had experienced microaggressions in medical school, including a number related to religion [11]. As this study occurred at the authors' home institution, it was felt that a survey question on this issue should be added to the HSCL's online assessment. Finally, the digital nature of this survey meant the team felt that questions related to online inclusivity should be addressed; recent literature highlights the importance of querying patrons' perceptions of library web pages for people with disabilities [12].

The survey (Appendix A) was migrated into Qualtrics by the HSCL's Undergraduate Fellow. The Fellow also added an attention check question (for which participants were asked to select a particular response) and reviewed the survey draft for inclusive language (e.g., non-gendered language, people-first, non-ableist language, and avoiding the use of slang or jargon). Demographic questions were added relating to student/faculty/staff status, college affiliation, and campus location. More personal demographic questions (age, sexual orientation, gender identity, whether international student/employee, race/ethnicity, first language, whether first generation college student, and caretaker status) were prefaced with text explaining why these questions were asked, and reminding participants that these questions were optional and could be skipped or responded to with “prefer not to answer.” These demographic questions were developed through feedback from groups with diverse identities, including the University of Florida's Presidential LGBTQ+ Advisory Committee, the Medical Library Association's Diversity and Inclusion Task Force, and HSCL's DEI Team. The survey was pilot tested by five Student Assistants, who suggested minor word changes to clarify meaning. The final version was submitted to the University of Florida's IRB-02 (Behavioral/Non-medical) and was approved as exempt on February 11, 2020.

The survey was launched on February 12, 2020, and was publicized through the library's website, social media (Twitter, Facebook), and emails from liaison librarians to their liaison groups. Personal emails were sent to representatives of target groups, such as Health Science Center (HSC) student affinity groups, the Disability Resource Center, the Office of Postdoctoral Affairs, and the HSC Diversity Liaisons Group which has DEI representatives from all six HSC colleges. The survey closed on April 29, 2020.

Two sub-groups of the research team, one for quantitative responses (three individuals) and one for qualitative responses (three individuals), analyzed the data. Quantitative data visualization was performed in Microsoft Excel and tests for significant differences among demographic groups were performed using the open-source statistical software JASP (version 0.14.1). Mann-Whitney U tests were used to compare means across two groups (e.g., caregiver or non-caregiver), and Kruskal-Wallis tests were used to compare means across more than two groups (e.g., female, male, or other response to gender). Non-parametric tests were used because the data was not normally distributed but was left-skewed. Two responses were eliminated from quantitative data analysis because participants selected “Strongly Agree” for every question, including the attention check question (which read “Please select Somewhat Disagree for this question”) and question 17 (which was negatively-worded, with a lower score indicating a positive response). For the purpose of analysis and to minimize the effects of small sample sizes, any demographic category with fewer than eight responses was grouped together, typically under the category of “Other.” For example, three individuals self-reported as lesbian, three as gay, and one as queer; these were grouped together during analysis as “Other LGBTQ+” because no individual category reached the threshold of eight responses.

The qualitative survey data was coded inductively by three of the researchers, using Braun and Clarke's thematic analysis as a basis [13]. First, two researchers individually identified preliminary patterns (codes) from the data. Through discussion, a list of consensus-based codes was created. Next, the qualitative data was coded separately by both researchers and synthesized into likely themes. Lastly, five themes were finalized by discussing definitions and reaching a mutual agreement. During the manual analysis process, the creation of codes and themes is inevitably influenced by the researchers' professional and personal identities and perspectives. As such, the final themes were reviewed and confirmed by a third researcher, who used NVivo to autocode respondents' comments to ensure that no potential themes were missed.

## RESULTS

Objective question responses were received from 101 individuals, alongside 24 open-ended responses.

### Demographics

Of the 101 individuals who responded to the survey, 21.3% were undergraduate students, 25.3% were professional students (i.e., MD, DVM, DMD, etc.), 13.3% were graduate students (i.e., PhD, masters), 1.3% were postdoctoral associates or fellows, 22.7% were faculty members, 20.0% were staff members, and 1.3% had another status at the university. In terms of gender, 74.7% of respondents identified as female, 1.3% as gender neutral/agender, 2.7% as genderqueer, 22.7% as male, and 4.0% preferred to self-describe. Participants were asked whether they were international students or employees; 13.3% were and 86.7% were not. In terms of race and ethnicity, 6.7% identified as Asian, 5.3% as Black or African American, 20.0% as Hispanic or Latinx, 2.7% as Middle Eastern or North African, 68.0% as white, 2.7% preferred to self-describe, and 5.3% preferred not to answer. Participants were asked whether or not English was their native language/first language; for 80.0% of respondents it was and for 20.0% of respondents, it was not. Other native languages reported included Spanish (62% of those whose first language was not English), Portuguese (15%), Arabic (8%), German (8%), and Mandarin (8%). Respondents were asked whether they were the first generation in their families to attend college or university; 25.3% were, 73.3% were not, and 1.3% preferred not to answer. In terms of sexual orientation, 13.5% identified as bisexual, 4.1% as gay, 70.3% as heterosexual, 4.1% as lesbian, 1.4% as queer, 1.4% preferred to self-describe, and 5.4% preferred not to answer. Participants were asked whether they had any caregiving responsibilities; 17.3% were caring for a child or children, 6.7% were caring for one or more parents, 1.3% were caring for a spouse, 73.3% were not caregivers, 5.3% listed another type of caregiving, and 2.7% preferred not to answer. For most demographic questions, participants could select multiple responses; the exceptions from the above list were for the questions about international students/employees, English as a native/first language, and first generation to college/university. Full demographic information of respondents is available in Appendix B.

### Quantitative Results

Overall, the quantitative survey responses were relatively positive, with means for each question ranging from 3.72 to 4.82 (with 5 = Strongly agree, 4 = Somewhat agree, 3 = Neither agree nor disagree, 2 = Somewhat disagree, 1 = Strongly disagree). Exceptions to this range were question number 12 (2.05), the attention check question, and question number 17 (1.88), which was negatively-worded, with a lower score indicating a positive response. The three questions with the highest mean responses were: “I feel I am treated with respect by this library's staff” (4.82); “I feel welcome to use this library's services” (4.81); and “I feel this library is a physically safe space for people of all backgrounds” (4.66). Beyond questions number 12 and number 17 described above, the three questions with the lowest mean responses were: “I am satisfied with this library's services for people whose native language is not English” (3.72); “I feel this library reliably meets the needs of individuals with disabilities” (3.89); and “I feel this library is a welcoming environment for families” (3.89). See Table 1 for the mean scores and standard deviations for all 18 questions.

**Table 1:** Mean response for each Likert question. Response options were: 1=Strongly disagree, 2=Somewhat disagree, 3=Neither agree nor disagree, 4=Somewhat agree, 5=Agree. Question number 12 was the attention check question and question number 17 was negatively-worded, with a lower score indicating a positive response.

Q #	Question	Average Response	Standard Deviation
1	I feel welcome to use this library's services.	4.8	0.4
2	I feel I belong at this library.	4.6	0.8
3	I feel I am treated with respect by this library's staff.	4.8	0.4
4	I feel I am treated with respect by other visitors at this library.	4.4	0.8
5	I feel this library is an inclusive physical space.	4.6	0.8
6	I feel this library is a physically safe place for people of all backgrounds.	4.7	0.7
7	I feel this library is an emotionally safe space.	4.5	0.8
8	I feel this library has an inclusive digital presence (website/social media).	4.5	0.8
9	I am satisfied with this library's services for people whose native language is not English.	3.8	1.0
10	I feel this library reliably meets the needs of individuals with disabilities.	3.9	1.1
11	I feel this library is a welcoming environment for families.	3.9	1.1
12	Please select Somewhat Disagree for this question.	2.1	0.6
13	I feel this library's services are fair and equitable.	4.5	0.8
14	I feel this library provides relevant services for diverse populations.	4.4	0.9
15	I feel that this library's staff will take appropriate action in response to incidents of discrimination within an acceptable period of time.	4.3	0.9
16	I feel this library is a welcoming environment for all religious and spiritual practices.	4.2	1.0
17	I feel afraid that when I visit the library others will think I lack the knowledge and/or skills to be there.	2.0	1.4
18	I feel this library demonstrates a strong commitment to diversity, equity, and inclusion.	4.3	0.9

For each of the 17 substantive questions in the online survey (excluding the attention check question), the authors tested whether there were different mean responses among demographic groups. There were relatively few statistically significant differences among demographic groups' responses to these questions. Those questions where one or more demographic categories showed statistically significant differences in response are summarized in Table 2.

**Table 2:** Questions which had statistically significantly different responses across one or more demographic group(s).

Question/Demographic group	Mean response	Statistical Test	Test value	p-value
I feel I belong at this library.				
International Student/Employee	4.00	Mann Whitney	459	<0.00
Not International Student/Employee	4.63			
I feel this library is a physically safe place for people of all backgrounds.				
Bisexual	4.20	Kruskal-Wallis	5.938	0.05
Other LGBTQ+	4.86			
Heterosexual	4.73			
I feel this library is an emotionally safe space.				
International Student/Employee	3.90	Mann Whitney	429.5	0.03
Not International Student/Employee	4.59			
Caregiver	4.07	Mann Whitney	302	0.05
Not a Caregiver	4.59			
I am satisfied with this library's services for people whose native language is not English.				
Caregiver	3.20	Mann Whitney	274.5	0.03
Not a Caregiver	3.90			
I feel this library provides relevant services for diverse populations.				
White	4.48	Kruskal-Wallis	9.72	0.01
Hispanic or Latinx	4.47			
Other Non White	3.50			
I feel this library is a welcoming environment for all religious and spiritual practices.				
Undergraduate Student	3.31	Kruskal-Wallis	15.04	0.01
Graduate Student	4.44			
Professional Student	4.71			
Other	4.29			
Staff Member	4.42			
Faculty Member	3.89			

### Qualitative Results

Twenty-four responses to the open-ended questions were received. Through thematic analysis the researchers identified five themes defined as follows:

1. **Reference and Liaison Services**: Reference assistance by librarians or information desk staff, library instruction, mediated literature searching, and circulation.
2. **Physical Space**: Comfort and accessibility of the physical library, including seating, door access, elevator access, group/quiet space, lighting, food preparation facilities, and temperature.
3. **Digital Space**: Website DEI-related content, website accessibility, availability of synchronous online reference and instruction services.
4. **DEI Initiatives**: Physical and digital library initiatives related to diversity, equity, and inclusion.
5. **Library Climate**: The library's collective atmosphere, including but not limited to interactions with library employees.

#### Theme 1: Reference and Liaison Services

Two respondents commented on the availability of resources for patrons who speak English as their second language. One person recommended having “librarian or library staff available to help locate and navigate resources or references specifically in non-English languages”, while another mentioned a need for “resource[s] for interpreters”. Perceived inequities in mediated literature searching services for tenured versus non-tenured faculty were a concern, with one respondent stating:

“I feel that the librarian is helpful to show us how to do literature searches but only performs them for tenure faculty and administration when asked. There have been times when I requested the literature search for a project/manuscript I was writing only to have returned to me a list of term[s] for the search…I have witnessed [the librarian] eagerly performing these searches for tenured and administrative faculty. It is extremely discouraging. Therefore, I do not bother her anymore.”

#### Theme 2: Physical Space

The predominant issue related to space was accessibility for people with disabilities, specifically in relation to seating, door access, and elevator access. Respondents voiced the following concerns and recommendations:

“As a person with a chronic illness, the frequent issues with the escalator make it difficult for me to get up to the main floor. Additionally, searching for a seat can be really hard on my body. Maybe consider having seats near the elevators reserved for people with disabilities.”

“Open the doors to the third floor during open hours so that wheelchairs and people that need to use the elevator can use that floor after 5 p.m.”

Patrons' perceptions of comfort in the library were influenced by the physical space. Requests made by participants included a microwave, more print books, a quiet or meditation space, and better temperature regulation in study rooms which were considered “stifling, as they overheat due to broken A/C”. Improved lighting was also mentioned:

“The lighting on the third floor is not conducive to learning. There is no natural light and although there are individual lights at each desk this causes unnecessary eye strain.”

#### Theme 3: Digital Space

Feedback highlighted two distinct information needs relating to accessibility, the first was additional website content on resources for people with visible and invisible disabilities, and the second was a need for information for distance learners on synchronous and asynchronous library services:

“If [the] library has special software, equipment, services for people with disabilities (especially invisible ones), [it would be] nice to have those listed in an easy-find accessible webpage.”

“Distance students, online students, students that do not live in [town name] like me, need different online support and help. I'd like to see more online or chat or availability in some way for distance and online DNP college of nursing students.”

An additional more generalized comment about the website was that it “can be difficult to navigate, which can decrease accessibility”.

#### Theme 4: DEI Initiatives

Perceptions of the library's DEI initiatives were overwhelmingly positive, with most respondents indicating that they value DEI efforts as well as providing ideas for the future. LGBTQ+ inclusivity initiatives were specifically mentioned by one participant, who stated that they felt “very welcomed by the Pride flag on the sign at the entrance” and that “the all gender bathrooms are so nice to see…you're doing a great job ensuring that people feel comfortable.” One respondent suggested that the library should have a large world map, so that employees and patrons can share where they are from alongside the different languages they speak. Additionally, the library's diverse exhibitions were praised:

“It's amazing to see all the interesting displays you put on. The Wartime Disability exhibit was exceptionally good, and the graphic novel competition was an innovative way to reach a wider audience with medical resources.”

However, one negative comment on the library's DEI initiatives was received, with a respondent stating “just be a library, what's with all this diversity stuff”.

#### Theme 5: Library Climate

The final theme encompassed participants' emotions, perceptions, and experiences in relation to the library's atmosphere, including feelings stemming from interactions with library employees. Positive feedback included comments on the liaison librarian program, the staff, and the welcoming and inclusive environment:

“I feel the library is very inclusive.”

“It's certainly the most welcome I feel anywhere on campus.”

“Best library on the planet!”

In contrast, negative feedback was received regarding one participant's experience with a library security guard and library staff, which strongly suggests that this patron was subject to racial and religious profiling:

“When I have worn a religious head covering, it is assumed that I do not know how to speak English or that I am not a student. Once, I had a security guard search my bag because “you look like you might have a bomb”.”

“Please educate your staff and security guards on stereotypes and make them aware that they may have incorrect biases against people.”

## DISCUSSION

### Quantitative Discussion

Overall, the quantitative findings showed a largely positive perception of the library's DEI climate and inclusivity, with only three questions having an average response below 4.0 (“Somewhat agree”). Questions about feeling welcome to use library services and feeling that the library is a physically safe space were among those with the highest responses; similarly, Stewart et al. found in their survey of African American students that interactions with the library as place contributed significantly to students' perceptions of how welcoming the library was [14].

The three lower-scoring questions indicate that areas for improvement include services for people whose native language is not English, for individuals with disabilities, and for families. While no equivalent studies have been done specifically in libraries, a broader climate survey conducted at Case Western Reserve University (CWRU) showed a similar response regarding the accessibility of campus to individuals with physical disabilities [6]. These responses in particular, in combination with the qualitative data, provide direction for future action at HSCL. Although the number of respondents was small, the survey results suggest that caregivers, particularly caregivers of children, do not find the library to be the most welcoming place for families. The question of academic library inclusion for students who are caregivers has not been well researched in the literature, but some libraries are creating dedicated family spaces [15, 16]. Professional students who make up a significant portion of HSCL's users are generally older than traditional undergraduates, which means that services to caregivers are especially important. A goal is to create space that makes it possible for parents to use HSCL's spaces and services more successfully.

Several questions showed significantly different responses among demographic groups. International students and employees responded less positively to the statement “I feel I belong at this library.” While a similar response was found in the CWRU data, where international respondents were slightly less likely to find their institution a comfortable place [6], this differs notably from responses to a University of Michigan campus climate survey, in which foreign-born students had no significant differences in feelings of being valued and belonging at their institution [7]. Respondents who identified as bisexual responded less positively than other LGBTQ+ or heterosexual respondents to the question about feeling that the library is a physically safe space. To some extent, this mirrors the Michigan survey, in which LGBTQ+ students reported feeling that they had been discriminated against significantly more than heterosexual students, though the difference between bisexual as compared to other LGBTQ+ respondents is somewhat puzzling. Both international students/employees and caregivers responded less positively than their non-international and non-caregiving counterparts to the statement “I feel this library is an emotionally safe space.” Caregivers also responded more negatively to the statement “I am satisfied with this library's services for people whose native language is not English.”

For the question “I feel this library provides relevant services for diverse populations,” the only statistically significant differences were between the race/ethnicity subgroups. The means for Hispanic/Latinx and white responses were very similar, but the mean for other non-white races and ethnicities was significantly lower, indicating that they are experiencing the library differently. According to state and university level demographic data, Hispanic/Latinx students represent the second largest racial and ethnic group on campus as well as in Florida overall [17]. The responses suggest that the two most well-represented racial and ethnic groups are experiencing the library differently than groups in the minority. While our data is aggregated for non-white and non-Hispanic groups, other studies including Stewart et al. discussed above also found that Black students reported instances of feeling unwelcome and communicated areas of concern [14]. This is similar to the data from the CWRU study, which found that minority respondents were 10% less likely to feel that their institution offered ample ethnic/cultural programming [6]. Special effort should be taken to provide not only equitable, but inclusive services particularly for racial and ethnic groups whose interactions with library resources and staff could lessen their feeling of welcome.

For the question “I feel this library is a welcoming environment for all religious and spiritual practices,” there were significant differences between the status subgroups, with undergraduate students and faculty members less likely to agree with this statement than other sub-groups. These findings align with the CWRU data in some ways and differ in others; the CWRU data indicated that undergraduates were most likely (11%) to feel discriminated against based on religion/spiritual beliefs and faculty were least likely (3%) [6]. Without further analysis and study, we cannot posit reasons for these differences, but we can try various interventions and initiatives to address these perceptions.

### Qualitative Discussion

The qualitative findings indicate that the strengths of HSCL's digital and physical environments in relation to DEI, as perceived by survey participants, are the library's exhibitions and programming, as well as its LGBTQ+ inclusivity initiatives (including all-gender bathrooms and the digital Pride flag). Additionally, feedback regarding the library's atmosphere was mostly positive, with respondents stating they felt welcome and included.

In contrast, a number of opportunities for enhancement became evident. One potential area for improvement is non-English language resources, particularly the availability of employees to assist with locating, navigating, and interpretation. Another area is perceived inequities between mediated literature searching services for tenured versus non-tenured faculty, which implies that clearer procedural correspondence from liaison librarians may be needed. Specific needs were highlighted regarding lack of accessibility to some physical spaces for people with disabilities, such as reliable elevator access, after-hours door access to the library's third floor, and seating closer to the entrances. Patrons' physical comfort could possibly be improved through enhancing or promoting existing quiet spaces, meditation spaces, lighting options, and air conditioning. In terms of HSCL's website, helpful content additions would encompass resources for people with visible and invisible disabilities, as well as more detailed information on library services available for distance learners. For DEI initiatives, a world map indicating where people have traveled from would likely be appreciated by patrons. Finally, an integral need to educate library employees and the wider university community about the importance of DEI on a continual basis was identified - a need that is very clearly reflected through the concerning racial and religious profiling incident recounted by one respondent, as well as the response from the participant who stated: “just be a library, what's with all this diversity stuff”.

### Implications and Current Initiatives

The DEI Team has begun addressing the needs and concerns highlighted by the survey results. Regarding feedback about HSCL's physical space, one of the most pressing issues is accessibility for people with mobility-related disabilities. The Team was able to advocate for the building's facilities personnel to fix the ADA door access buttons that had been working inconsistently for several months. Although the building's elevator is outside of the library's control, facilities workers have fortuitously repaired it by the time of this article's writing. For security reasons the third-floor doors to the library remain locked after hours; however, two wheelchair-accessible quiet study spaces are available on the library's second floor which is open to eligible library card holders 24/7; this information has been added to HSCL's “Patrons with Disabilities” webpage [18]. The provision of reserved ADA seating located near the library entrances is being investigated. Participants' comments on the need for temperature regulation likely stemmed from a broken air handler during the study timeframe, resulting in hot temperatures in study rooms in particular. In response to patrons' articulated needs for space changes, HSCL is promoting its existing quiet space on the third floor as well as its meditation sessions. The library is also exploring the possibility of purchasing sunlight lamps, creating a meditation room, and designating a family area for patrons with children.

In terms of HSCL's online services and website, additions to the “Patrons with Disabilities” webpage include a detailed map of accessible entrances to the library building, information on proxy borrowing for patrons registered with UF's Disability Resource Center, and available assistive technology. Specific messaging was placed on the website and promoted through social media concerning the availability of virtual services for distance learners; this became particularly important in 2020, as the majority of patrons became distance learners during the height of the COVID-19 pandemic. A LibGuide for distance learners is currently being updated and will be promoted. Additionally, the DEI Team is now conducting a language inclusivity review of HSCL's website and LibGuides.

The Team is also considering how best to respond to the somewhat negative responses about services for individuals whose first language is not English and the somewhat lower responses among international students and personnel to two questions. The Team is planning to conduct focus groups with non-native English speakers to identify their specific barriers and elicit suggestions on changes to library services and resources that would serve their needs. Services suggested in the literature that we may investigate include providing resources related to language learning, keeping non-English language newspapers and magazines in the library, holding orientations and instructional sessions specifically for international learners, having a designated point of contact for international learners, and continuing staff training on cultural awareness [19]. The qualitative results showed that physical representations of inclusivity are important to patrons. The Team is considering that a map celebrating the Health Science Center's global community might be displayed in a permanent fashion, rather than solely during international education week, as was done previously. HSCL will certainly continue to provide welcoming signs in multiple languages. Additionally, HSCL will continue to collaborate with other campus entities that focus on internationalization. UF has a robust International Center, and the broader UF libraries recently created a Global Engagement Committee, whose membership includes one member from HSCL's DEI Team. HSCL has consistently partnered with both groups to celebrate international education week, hosting programming including photographic exhibits and speakers, and will actively pursue new partnerships as they become available.

The Team's response to the profiling incident is multifaceted. While the feedback from this patron implies that they experienced these issues at another campus library (due to the mention of an escalator, which the HSCL does not have), the Team is dedicated to continually improving the DEI climate to meet the ever-evolving needs of patrons and the local community. First, a Code of Conduct has been created that applies to all library employees and patrons using physical and digital spaces and services. The Code of Conduct states that “HSCL seeks to provide a safe, inclusive, and supportive environment that fosters mutual respect for all people” [20]. It sets out behavioral expectations for everyone, provides details for whom to contact concerning violations, and lists step-by-step responses to infractions. Instances of profiling, stereotyping, or any other exclusionary words, actions, and behavior will not be tolerated and will be addressed directly. Second, the DEI Team reached out to library administration to ensure that security guards are required to undergo campus DEI training. Third, the Team continues to develop and host trainings, workshops, and other events to engage library employees, current patrons, and prospective patrons in DEI conversations. Two recent examples are collaborations with the College of Medicine during their 2021 Celebration of Diversity Week. HSCL organized the panel discussion “Honoring LGBTQIA+ Health Stories”, with the goal of educating future health providers on the health needs of LGBTQIA+ communities through providing an opportunity for panelists to share their experiences as LGBTQIA+ health providers who are also patients. The Team also hosted a Race Card Wall as part of the Race Card Project, which is a global initiative [21]. The goal of the Race Card Project is to create an educational conversation around race, through sharing individuals' stories, learning from each other, and gaining a deeper understanding of society.

Finally, the Team interpreted the response from the participant who stated “just be a library, what's with all this diversity stuff”, as evidence that stakeholders do not always understand why libraries play an integral role in DEI initiatives. Libraries should therefore continually strive to educate on DEI issues in all spaces through outreach and community engagement. While this negative feedback was jarring, the Team feels that this type of response only emphasizes the need for ongoing DEI initiatives in all spaces.

## STUDY LIMITATIONS

In comparison to the HappyOrNot pilot, the DEI Team felt that the online survey facilitated a deeper understanding of the DEI needs and expectations of different patron populations. However, there were some limitations. While the team had hoped to be able to make comparisons among groups with specific identities (for example, respondents whose first language was not English and are also members of other marginalized groups versus English speakers from those same groups), the number of responses were too few to make such comparisons of subpopulations meaningful. Given the size of the population of the Health Science Center, responses overall were relatively low. A probable reason was that the final five weeks of the survey ran when the library had closed due to COVID-19 and university operations were in flux. This low turnout and the fact that the team used a convenience sample suggest that the results may not be generalizable. Another limitation was that the Team realized they had not added an option for individuals to self-identify with one or more disabilities; this could have brought more context to the responses regarding whether the library meets the needs of people with disabilities. Because the survey was widely distributed, it is possible that some responses referred to a different campus library. If the survey were administered again, respondents would be provided with an opportunity to indicate which library or libraries their responses refer to. Finally, like any study that relies on surveys, the results reported here reflect a specific snapshot in time. The survey ran prior to the events of the summer of 2020, a time of reflection on and protest for racial justice. While the authors do not consider this a limitation of the study, it is intriguing to consider how responses to questions, particularly those related to safety and equity, may have differed if posed in the fall of 2020.

## CONCLUSION

Overall, the survey provided useful data that highlights the library's strengths in relation to DEI and the HSCL's digital and physical environments. While feedback on service gaps and other potential improvements was received, this was welcomed and viewed as constructive. In light of this, the DEI Team is dedicated to continually improving the climate for library patrons and UF's wider communities. The Team recognizes that DEI work is an ongoing process and looks forward to supporting patrons' educational pursuits through recognizing and meeting diverse needs.

## Data Availability

Only aggregated data is available to ensure that any survey respondents with unique demographic characteristics remain unidentifiable.

## SUPPLEMENTAL FILES

**Appendix A:** Survey Questions

**Appendix B:** Full Demographic Results
