# Supplementary material for: Identifying diversity, equity, and inclusion enhancement opportunities through an online mixed methods library survey
Source: J Med Libr Assoc. 2022 Oct 1;110(4):438–48. doi: 10.5195/jmla.2022.1436 (PMC10124613; doi:10.5195/jmla.2022.1436)
Supplement: Supplementary file 1 — Appendix A: Survey Questions [file jmla-110-4-438-s01.pdf]

Appendix A: Survey Questions

(bolded questions were modified directly from the HappyOrNot pilot)

Please indicate your level of disagreement or agreement with each statement [strongly agree, somewhat agree, neither agree nor disagree, somewhat disagree, strongly disagree].

1. **I feel welcome to use this library's services.**
2. I feel I belong at this library.
3. **I feel I am treated with respect by this library's staff.**
4. **I feel I am treated with respect by other visitors at this library.**
5. **I feel this library is an inclusive physical space.**
6. **I feel this library is a physically safe place for people of all backgrounds.**
7. **I feel this library is an emotionally safe space.**
8. I feel this library has an inclusive digital presence (website/social media).
9. **I am satisfied with this library's services for people whose native language is not English.**
10. **I feel this library reliably meets the needs of individuals with disabilities.**
11. **I feel this library is a welcoming environment for families.**
12. Please select Somewhat Disagree for this question.
13. **I feel this library's services are fair and equitable.**
14. I feel this library provides relevant services for diverse populations.
15. **I feel that this library's staff will take appropriate action in response to incidents of discrimination within an acceptable period of time.**
16. I feel this library is a welcoming environment for all religious and spiritual practices.
17. I feel afraid that when I visit the library others will think I lack the knowledge and/or skills to be there.
18. **I feel this library demonstrates a strong commitment to diversity, equity, and inclusion.**

Have you ever considered not using library services because you felt unwelcome?

- Yes
- No [If no, skip next question]

Please tell us why, so that we can improve. [Open response].

Please tell us how the HSC Library could improve in the areas listed above. [Open response].

What is your status at UF? (Check all that apply)

- Undergraduate Student (BHS, BSN, etc.)
- Professional Student (MD, PharmD, DVM, DDS, PA, etc.)
- Graduate Student (Master, PhD, etc.)

- Resident or Fellow
- Post-Doctoral Associate/Fellow
- Faculty Member
- Staff Member
- UF guest/general public
- Other (please specify)

What is your college affiliation? (Check all that apply)
- Medicine
- Nursing
- Pharmacy
- Public Health and Health Professions
- Veterinary Medicine
- Other (please specify)

On which campus are you located? (Check all that apply)
- Jacksonville
- Orlando
- Online/Distance
- Pensacola
- Other (please specify)

Some of the following demographic questions are more sensitive in nature. We are asking them because we are committed to ensuring that library users belonging to underserved groups have positive experiences of the library and its services. These questions are completely optional, you may choose not to respond by either skipping the question or selecting “prefer not to answer” as your response for each question.

Please select the age range which applies to you.
- 19 and under
- 20-29
- 30-39
- 40-49
- 50-59
- 60 and over
- Prefer not to answer

How do you identify? (Check all that apply)
- Female
- Gender Neutral/Agender

- Genderqueer (umbrella term for bigender, third gender, gender non-conforming, gender-fluid, non-binary, two-spirit)
- Male
- Prefer to self describe [open response]
- Prefer not to answer

Would you describe yourself as transgender?
- Yes
- No
- Prefer to self describe [open response]
- Prefer not to answer

Are you an international student or employee?
- Yes
- No
- Prefer to self describe [open response]
- Prefer not to answer

With which racial or ethnic group(s) do you identify? (Check all that apply)
- Asian
- Black or African American
- Hispanic or Latinx
- Indigenous American or Indigenous Alaskan
- Indigenous Hawaiian or Pacific Islander
- Middle Eastern or North African
- White
- Prefer to self describe [open response]
- Prefer not to answer

Is English your native language/first language?
- Yes
- No [If no, skip to question 15]

What is your native language/first language? [Open response]

Do you identify as a first-generation student (the first in your family to attend college/university)?
- Yes
- No
- Prefer not to answer

How do you describe your sexual orientation? (Check all that apply)

- Bisexual
- Gay
- Heterosexual
- Lesbian
- Pansexual
- Queer
- Questioning
- Prefer to self describe [open response]
- Prefer not to answer

Are you a caregiver? (Check all that apply)

- Caring for child/children
- Caring for parent/s
- Caring for spouse
- Not a caregiver
- Other [open response]
- Prefer not to answer
