# Supplementary material for: Identifying diversity, equity, and inclusion enhancement opportunities through an online mixed methods library survey
Source: J Med Libr Assoc. 2022 Oct 1;110(4):438–48. doi: 10.5195/jmla.2022.1436 (PMC10124613; doi:10.5195/jmla.2022.1436)
Supplement: Supplementary file 2 — Appendix B: Full Demographic Results [file jmla-110-4-438-s02.pdf]

Appendix B: Full Demographic Results

Demographic	Value	Number	Percentage
Status at UF*	Undergraduate Student	16	21.3%
	Professional Student	19	25.3%
	Graduate Student	10	13.3%
	Resident or Fellow	0	0.0%
	Post-Doctoral Associate/Fellow	1	1.3%
	Faculty Member	17	22.7%
	Staff Member	15	20.0%
	UF guest/general public	0	0.0%
	Other	1	1.3%
College*	Dentistry	13	17.6%
	Medicine	9	12.2%
	Nursing	7	9.5%
	Pharmacy	3	4.1%
	Public Health and Health Professions	24	32.4%
	Veterinary Medicine	7	9.5%
	Other	14	18.9%
Campus	Gainesville	73	97.3%
	Jacksonville	0	0.0%
	Orlando	0	0.0%
	Online/Distance	2	2.7%
	Pensacola	0	0.0%
	Other	0	0.0%
Age	19 and under	4	5.3%
	20-29	35	46.7%
	30-39	15	20.0%
	40-49	4	5.3%

	50-59	7	9.3%
	60 and over	9	12.0%
	Prefer not to answer	1	1.3%
Gender*	Female	56	74.7%
	Gender Neutral/Agender	1	1.3%
	Genderqueer (umbrella term for bigender, third gender, gender non-conforming, gender-fluid, non-binary, two-spirit)	2	2.7%
	Male	17	22.7%
	Prefer to self describe	3	4.0%
	Prefer not to answer	0	0.0%
Gender identity	Transgender	1	1.3%
	Not transgender	73	97.3%
	Prefer to self describe	1	1.3%
	Prefer not to answer	0	0.0%
International Status	International Student/Employee	10	13.3%
	Not International Student/Employee	65	86.7%
	Prefer to self describe	0	0.0%
	Prefer not to answer	0	0.0%
Race/Ethnicity*	Asian	5	6.7%
	Black or African American	4	5.3%
	Hispanic or Latinx	15	20.0%
	Indigenous American or Indigenous Alaskan	0	0.0%
	Indigenous Hawaiian or Pacific Islander	0	0.0%
	Middle Eastern or North African	2	2.7%
	White	51	68.0%
	Prefer to self describe	2	2.7%
	Prefer not to answer	4	5.3%

Native language/first language	English	60	80.0%
	Not English	15	20.0%
First generation to college/ university	First Generation	19	25.3%
	Not First Generation	55	73.3%
	Prefer not to answer	1	1.3%
Sexual orientation*	Asexual	0	0.0%
	Bisexual	10	13.5%
	Gay	3	4.1%
	Heterosexual	52	70.3%
	Lesbian	3	4.1%
	Pansexual	0	0.0%
	Queer	1	1.4%
	Questioning	0	0.0%
	Prefer to self describe	1	1.4%
	Prefer not to answer	4	5.4%
Caregiving status*	Caring for child/children	13	17.3%
	Caring for parent/s	5	6.7%
	Caring for spouse	1	1.3%
	Not a caregiver	55	73.3%
	Other	4	5.3%
	Prefer not to answer	2	2.7%

* Indicates that the question stated, “Check all that apply.”
